# Diagnosis of gallbladder villous tubular adenoma with high-grade intraepithelial neoplasia via peroral endoscopic choledochoscopy: a rare case of an elderly patient

**DOI:** 10.1055/a-2772-5625

**Published:** 2026-01-28

**Authors:** Jianguo Zhang

**Affiliations:** 1 12465 Department of Biomedical Engineering, College of Future Technology, Peking University, Beijing, China; 2 Department of Gastroenterology, Aviation General Hospital of China Medical University, Beijing, China

Gallbladder adenomas can be classified into three types based on their growth patterns: tubular, papillary, and tubulopapillary 1 . Ultrasound is usually the preferred imaging modality for diagnosing gallbladder diseases. However, it is difficult to differentiate adenomas from other gallbladder polyps 2 3 . Gallbladder villous tubular adenomas with high-grade intraepithelial neoplasia are carcinoma in situ in pathology 1 . Those with a clear diagnosis need surgical treatment. Therefore, accurate diagnosis of the lesion is crucial for treatment.

**Fig. 1:**
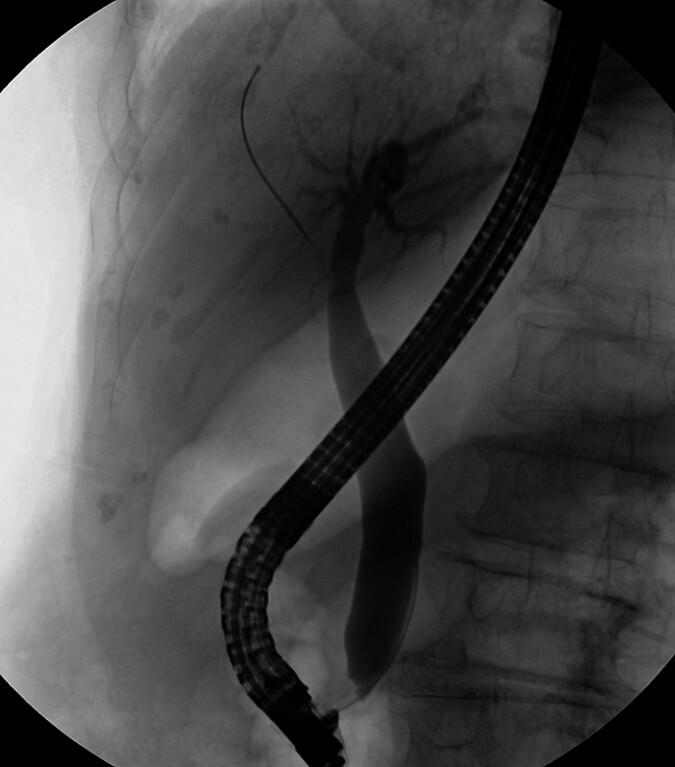
An X-ray image taken during ERCP. Dilation of the common bile duct was observed. ERCP, endoscopic retrograde cholangiopancreatography.

**Fig. 2:**
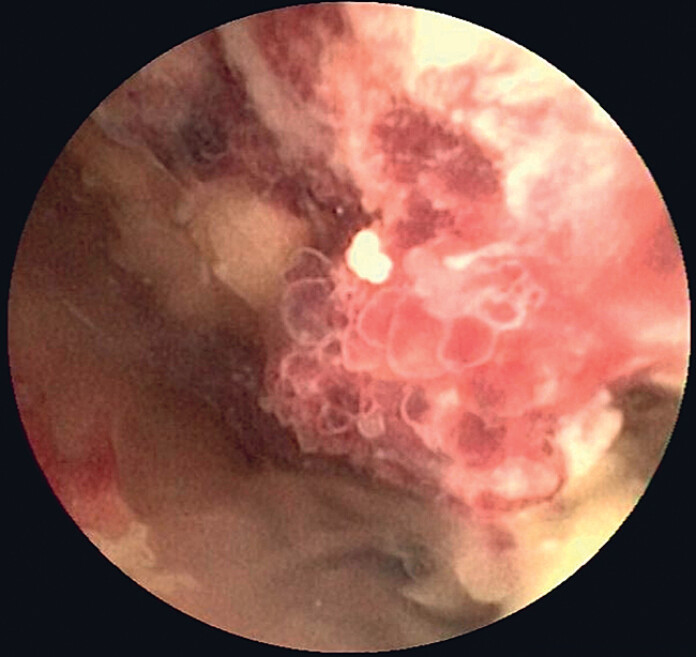
Villous tubular adenoma observed under direct vision.

**Fig. 3:**
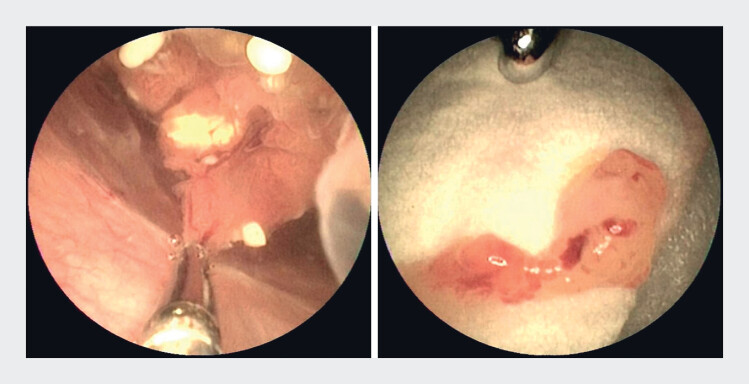
**a** Biopsy under direct vision. **b** A macroscopic view of the biopsy lesion.

This case report presents a rare case of a 90-year-old woman who was admitted to the hospital after a preliminary diagnosis of gallbladder space-occupying lesions. We first performed endoscopic retrograde cholangiopancreatography (ERCP) on the patient ( Fig. 1 ). Subsequently, biliary endoscopic sphincterotomy was performed via a small incision. A transoral cholangioscope was inserted, and no obvious abnormalities were found in the common bile duct and common hepatic duct. The opening of the cystic duct was located, and the gallbladder was entered along the cystic duct. A large, spherical, polyp-like mass was found in the fundus and body of the gallbladder ( Fig. 2 , Video 1 ). The surface of the mass was mostly smooth and regular, with a villous, coral-like appearance. Focal congestion, redness, and erosion were observed. A biopsy was taken under direct visualization using a transoral cholangioscopy ( Fig. 3 ). A small amount of bleeding occurred at the biopsy site, which stopped spontaneously with epinephrine saline spray. The pathology report showed the mass to be a tubular adenoma of the gallbladder with focal high-grade intraepithelial neoplasia, confirming the diagnosis of early gallbladder cancer. The patient subsequently underwent surgical intervention, specifically a laparoscopic cholecystectomy. The patient was discharged smoothly without complications.

A transoral cholangioscope was used to access the gallbladder to directly visualize and biopsy the gallbladder villous tubular adenoma.Video 1

This case demonstrates that endoscopic access to the gallbladder via natural orifices provides an effective minimally invasive diagnostic method for gallbladder lesions. Further research is needed to validate its role in accurately diagnosing gallbladder diseases.

Endoscopy_UCTN_Code_CCL_1AZ_2AN
